# Supplementary material for: Reconciling Mining with the Conservation of Cave Biodiversity: A Quantitative Baseline to Help Establish Conservation Priorities
Source: PLoS One. 2016 Dec 20;11(12):e0168348. doi: 10.1371/journal.pone.0168348 (PMC5173368; doi:10.1371/journal.pone.0168348)
Supplement: S1 Dataset — (ZIP) [file pone.0168348.s002.zip › Taxa/Serra Norte/SN_2013/N4WS/Lista N4WS-36.pdf]

CAVIDADE N4WS-0036

Classe	Ordem	Fam/Outros	Gên/Outros	Espécie	S-U	U-S	Zon.
Arachnida	Amblypygi	Phrynidae	*Heterophrynus*	*longicornis*	1	2	E
Arachnida	Araneae	Corinnidae		jovem	1		E
Arachnida	Araneae	Mysmenidae	*Microdipoena*	sp.1	X		E
Arachnida	Araneae	Pholcidae	*Mesabolivar*	*aurantiacus*		X	E
Arachnida	Araneae	Pholcidae	*Mesabolivar*	*eberhard*		X	E
Arachnida	Araneae	Pholcidae		jovem	X		E
Arachnida	Araneae	Theridiidae	*Theridion*	sp.1		X	E
Arachnida	Araneae	Theridiosomatidae		jovem		X	E
Insecta	Coleoptera	Lampyridae		jovem		X	E
Insecta	Diptera	Cecidomyiidae	Cecidomyiinae	sp.	X	X	E
Insecta	Diptera	Ceratopogonidae		sp.	X		E
Insecta	Diptera	Culicidae	*Culex*	sp.		X	E
Insecta	Diptera	Keroplatidae	*Macrocera*	sp.		X	E
Insecta	Diptera	Psychodidae	*Sciopemyia*	*sordellii*	X	X	E
Insecta	Diptera	Tipulidae	Tipulinae	sp.	X	X	E
Insecta	Homoptera	Cixiidae		sp.4		X	E
Insecta	Hymenoptera	Formicidae	*Pheidole*	sp.2		X	E
Insecta	Hymenoptera	Formicidae	*Pheidole*	sp.3		X	E
Insecta	Hymenoptera	Formicidae	*Solenopsis*	sp.2		X	E
Insecta	Isoptera	Termitidae	*Nasutitermes*	sp.	X	X	E
Insecta	Lepidoptera	Noctuoidea		sp.2		X	E
Insecta	Lepidoptera	Tineoidea		sp.6		X	E
Insecta	Orthoptera	Phalangopsidae	*Paraclodes*	sp.		2	E
Insecta	Orthoptera	Phalangopsidae	*Phalangopsis*	sp.	2	25	E
Malacostraca	Isopoda	Armadillidae		sp.1		X	E
Mammalia	Chiroptera	Emballonuridae	*Peropteryx*	sp.		3	E
